# Communicating wisely: teaching residents to communicate effectively with patients and caregivers about unnecessary tests

**DOI:** 10.1186/s12909-017-1086-x

**Published:** 2017-12-11

**Authors:** Geetha Mukerji, Adina Weinerman, Sarah Schwartz, Adelle Atkinson, Lynfa Stroud, Brian M. Wong

**Affiliations:** 1 Department of Medicine, University of Toronto and Women’s College Hospital Institute of Health Systems Solutions and Virtual Care, 76 Grenville Street, Room 3414, Toronto, ON M5S 1B2 Canada; 2 Department of Medicine, University of Toronto and Sunnybrook Health Sciences Centre, Toronto, ON Canada; 3 Department of Pediatrics, University of Toronto and Hospital for Sick Children, Toronto, ON Canada; 4 Department of Pediatrics, University of Toronto and Hospital for Sick Children, Toronto, ON Canada; 5 Department of Medicine, University of Toronto and Sunnybrook Health Sciences Centre, Toronto, ON Canada; 6 Department of Medicine, Sunnybrook Health Sciences Centre, University of Toronto, Toronto, ON Canada; 7 Centre for Quality Improvement and Patient Safety, University of Toronto, Toronto, ON Canada

**Keywords:** Resource stewardship, Communication, Medical education, High-value care

## Abstract

**Background:**

With rising healthcare costs and a focus on quality, there is a growing need to promote resource stewardship in medical education. Physicians need to be able to communicate effectively with patients/caregivers seeking tests and treatments that are unnecessary.

This study aimed to evaluate the impact of an interactive workshop on residents’ knowledge of resource stewardship and communication skills when counseling patients/caregivers about requests for unnecessary testing.

**Methods:**

Participants were 83 Internal Medicine and Pediatrics residents at the University of Toronto in 2014–15. The evaluation compared resource stewardship knowledge and communication skills of 57 (69%) residents that attended the resource stewardship workshop to 26 residents (31%) who did not. Knowledge and communication skills assessment consisted of a written test and a structured assessment using standardized patient raters, respectively. A linear regression was applied to determine predictors of overall communication skills performance.

**Results:**

Workshop attendance resulted in better performance on the knowledge test (4.3 ± 1.9 vs. 3.1 ± 1.7 out of 8, *p* = 0.01), but not better performance on the communication skills assessment (4.1 ± 0.8 vs. 4.0 ± 0.9 out of 5, *p* = 0.56). Higher training level (*p* = 0.01) and knowledge test scores (*p* = 0.046) were independent predictors of better overall communication skills, after adjusting for gender, training level, workshop attendance, knowledge and self-reported prior feedback on communication skills.

**Conclusions:**

An interactive workshop can improve knowledge of resource stewardship, but improving communication skills with patients/caregivers about unnecessary testing may require additional training or reinforcement in the clinical learning environment. These teaching and assessment approaches can support the integration of education on resource stewardship into medical education.

**Electronic supplementary material:**

The online version of this article (10.1186/s12909-017-1086-x) contains supplementary material, which is available to authorized users.

## Background

Physicians have a professional obligation to ensure appropriate use of healthcare resources [1, 2]. Unnecessary tests and treatments that do not enhance medical care have significant implications for the well-being of patients as they can expose patients to potential harm including procedural complications, side effects such as radiation or nosocomial infection, and unnecessary follow-up testing, and can also have significant health system impact by contributing to rising healthcare costs [3–7]. Examples include imaging for uncomplicated headaches and lower back pain, computed tomography abdominal scan as an initial evaluation of a child with suspected appendicitis, pap smears for women who have had hysterectomies or are under the age of 21 or routine check-ups for healthy adults [8]. Yet, current training does not adequately prepare physicians to deliver high-value care [9]. In 2012, less than 15% of programs surveyed had a formal resource stewardship curriculum [10]. Furthermore, residents who trained in clinical environments with high resource utilization tend to utilize more resources in future practice [11–14]. Therefore, there is a critical need to develop teaching and assessment strategies for resource stewardship education [15].

One driver of healthcare resource overuse is patient/caregiver request for unnecessary tests/treatments [16, 17]. The Choosing Wisely® campaign promotes *conversations* between physicians and patients/caregivers about unnecessary tests/treatments as a key strategy to ensure the delivery of high-value care [18–20]. Developing effective communication skills to address patient preference is essential for educating about resource stewardship, as demonstrated by a shared decision-making program with patients that led to fewer unnecessary antibiotic prescriptions [21].

A recent systematic review identified a number of curricula that teach key concepts related to resource stewardship, and identified the following factors that contribute to successful learning of resource stewardship concepts: 1) effective transmission of knowledge related to healthcare costs, practice guidelines, benefits and harms of healthcare, and patient preferences/values; 2) facilitation of reflective practice, including provision of feedback on laboratory ordering or prescribing practices; and 3) creation of a supportive environment with appropriate role modeling to promote a culture of high-value, cost-conscious care [22]. While there are published examples of curricula focused on resource stewardship [23–25], only one of the included studies in the systematic review had a small component focused specifically on communication skills training related to unnecessary tests and treatments [25]. Our study aims to evaluate the impact of a resource stewardship workshop on residents’ knowledge of resource stewardship and communication skills when counseling patients/caregivers about requests for unnecessary testing.

## Methods

### Setting and participants

This study included Internal Medicine (IM) and Pediatric residents across 6 medical centers affiliated with the University of Toronto (UofT) in 2014–15. All third-year IM residents (*N* = 64) and first to third year Pediatric residents (*N* = 75) were eligible to participate. The resident inclusion criteria were based on how the individual program directors chose to implement the curriculum. The IM program targeted their third-year residents, whereas the pediatric program introduced the curriculum to first to third year residents during their academic half-days. There were no exclusion criteria. Prior to this study, there was no formal resource stewardship training. The UofT research ethics board approved this study.

### Resource stewardship workshop

The 2.5-h interactive workshop focused on developing communication skills to counsel patients/caregivers about unnecessary testing, and was delivered to IM and Pediatric residents during their respective academic half-days (Table 1). Attendance at academic half-days is strongly encouraged but not mandatory, and some cannot attend due to vacation, illness or being post-call. We reviewed the literature and solicited input from the Choosing Wisely Canada® leadership team to inform our workshop. Specifically, we incorporated content relating to overcoming barriers to high-value care from the Alliance for Academic Internal Medicine and the American College of Physicians *High Value Care* curriculum [26], elements from the *VALUE Framework* that teaches residents to assess whether an intervention provides value [27], and a framework for communicating with patients /caregivers who are requesting unnecessary tests/treatments taken from the Choosing Wisely® Communication Modules developed in partnership with Drexel University College of Medicine [28]. Members of the research team who were clinicians with an academic interest in promoting high-value care and medical educators with expertise in assessment tools were involved in development and delivery of the curriculum.

**Table 1:** Resource Stewardship Workshop Description

The 2.5 hour workshop included 4 sections:
1. An introductory didactic mini-lecture that focused on the rationale for resource stewardship, harms of overuse, the Choosing Wisely® campaign, and facilitators and barriers of resource stewardship. (slides available by request)
2. A trigger video from the Choosing Wisely® Physician Communication Modules [28] that demonstrates a structured communication framework to counsel patients/caregivers when they are seeking an unnecessary test. The framework includes 4 key components: i) provide clear recommendations; ii) elicit patient beliefs and concerns; iii) provide empathy, partnership and legitimation; and iv) confirm agreement [29].
3. A role-play exercise with peers in small group sessions to apply the framework to mock communication scenarios and receive feedback. (see supplementary files for sample OSCE scenarios and assessment tools that can be used for this exercise)
4. A large group facilitated discussion to promote reflection and discussion about the use of the structured communication framework during the role-play and lessons learned that residents should carry forward to real-world interactions

### Program evaluation

We applied the Kirkpatrick model to support the evaluation of educational outcomes [30]. The knowledge assessment was a delayed knowledge test and the skills assessment was a structured communication skills assessment station integrated into the IM and Pediatric annual objective structured clinical examination (OSCE) (both Level 2 Kirkpatrick outcomes). Both assessments occurred approximately 3 months after the workshop. Program directors would not agree to random allocation of residents to being exposed or not to the curriculum. Therefore, to evaluate the impact of curriculum exposure on knowledge and skills, we compared residents who attended our workshop to those who did not.

#### Delayed knowledge test

We developed multiple-choice and short-answer questions to assess the knowledge taught during the workshop. We piloted questions with residents and faculty with resource stewardship expertise and iteratively modified questions for content and readability [see Additional file 1].

We generated an overall score based on their answers to the one short answer question and seven multiple-choice questions (total potential score of 8). For the short answer question, two study authors (GM and AW) independently scored the response and resolved discrepancies by consensus. We counted unanswered questions as incorrect.

#### Structured communication skills assessment

We developed hypothetical scenarios based on published recommendations from the Choosing Wisely® campaign and input from study authors who have prior experience creating OSCE stations. The IM scenario was a patient with a history of mild aortic stenosis, without any worrisome symptoms or signs, who is requesting an unnecessary repeat echocardiogram [see Additional file 2]. The Pediatric scenario was a parent of an infant who had a simple febrile seizure and a normal neurological examination, who is requesting an unnecessary magnetic resonance imaging (MRI) brain [see Additional file 3]. We piloted these scenarios with faculty and resident volunteers to ensure that they were realistic, readable, and feasible to complete in 10 min. At the time of the structured assessment, each resident read the clinical scenario and then counseled the standardized patient (SP) (playing either the patient or caregiver) about their request. The SP then used a rating scale to score the resident’s performance immediately after the encounter. The study team members were not involved in completing the rating scale to assess communication skills.

We created a novel rating scale that mapped specifically to the communication skills construct that we sought to assess. The scale includes the five components (i.e., clear recommendations, elicit patient/caregiver concerns, empathy, confirm agreement and general communication skills) of the structured communication framework that was taught as part of our workshop [see Additional file 4]. The SP rated performance on each of the 5 components and also provided a global rating using a five-point Likert scale. The SP also scored individual sub-items within each component using a three-point scale (1-not done at all; 2-attempted but either not complete or not effective; and 3-excellent).

We chose to use SPs as opposed to faculty raters because we sought to assess a resident’s ability to communicate about unnecessary tests from the perspective of the patient/caregiver. There is also high inter-rater reliability between ratings of trained SPs and trained faculty to assess other types of communication skills such as disclosing medical errors [31]. To promote consistency of SP ratings, we used the same 3 SPs for all skills assessments. SP training occurred on two separate occasions for each scenario. During the first session, the SPs familiarized themselves with the rating scale and practiced using it by viewing the training videos used during the workshop. During the second session, they met with study investigators, and familiarized themselves with the scenario and practiced it in pilot interactions with a volunteer resident. This allowed SPs to clarify details regarding the scenario and items on the rating scale, and ensured that there was agreement between the ratings of the three SP raters. To establish inter-rater reliability of SP ratings for this tool, we audiotaped the assessments on the day of the OSCE and had a second trained SP independently rate resident communication skills.

We obtained informed consent from participating residents prior to their formative OSCE. The residents received no incentive to participate. In addition to the knowledge test and structured assessment data, we also collected baseline demographic characteristics from participating residents including gender and training level. We also asked residents about their prior experiences with communicating with patients/caregivers about unnecessary tests/treatments and to rate their own comfort and skills in this area, as well as whether they have ever received feedback on these skills.

### Statistical analysis

We summarized participant characteristics descriptively, using means and standard deviations for continuous data and count and percentages for categorical data. We calculated a Cronbach’s alpha to determine the internal consistency of the scale items on the communication skills assessment.

We compared knowledge test scores and global ratings on the structured communication skills assessment for residents who attended the workshop versus those who did not attend using Wilcoxon rank sum test to better account for the non-normal distribution of scores. We also calculated intraclass correlation coefficients (ICC) to assess inter-rater reliability of the communication skills assessment ratings. To identify predictors of overall performance on the structured assessment, we incorporated five pre-specified variables of interest (gender, training level, workshop attendance, delayed knowledge assessment scores and self-reported prior feedback on resource stewardship communication skills) into a mixed-effects linear regression analysis. The model accounted for the correlation among observations from the same residency training program due to possible differences in case difficulty. The analyses were carried out using SAS version 9.3 (SAS Institute, Cary, NC, USA).

## Results

Of the 111 eligible IM and Pediatric residents, 98 (88%) participated in the structured assessment, of which 83 (75%) provided consent to have their assessment data used for analysis. Of the 83 study participants, 57 (69%) attended the resource stewardship workshop. Table 2 summarizes participant characteristics stratified by workshop attendance. There were no significant differences in participant characteristics comparing residents who attended the workshop to those who did not.

**Table 2:** Participant characteristics by workshop attendance (*N* = 83)

Characteristic*	Not Attended (*n* = 26)	Attended Workshop (*n* = 57)
Gender		
Male	12 (46%)	22 (39%)
Female	14 (54%)	35 (61%)
Training Program		
Internal Medicine	10 (38%)	35 (61%)
Pediatrics	16 (62%)	22 (39%)
Training Level		
Post-graduate Year 1	3 (12%)	13 (23%)
Post-graduate Year 2	5 (19%)	4 (7%)
Post-graduate Year 3	18 (69%)	40 (70%)
Self-Reported Prior Experience Communicating with Patient/Caregiver Regarding Unnecessary Tests		
Yes	24 (96%)	54 (98%)
No	1 (4%)	1 (2%)
Self-Reported Prior Feedback Received on Communication with Patient/Caregiver Regarding Unnecessary Tests		
Yes	18 (75%)	37 (67%)
No	6 (25%)	18 (33%)
Self-Reported Comfort with Communicating with Patient/Caregiver Regarding Unnecessary Tests		
Above average	12 (52%)	24 (44%)
Average	9 (39%)	29 (53%)
Below average	2 (9%)	2 (4%)
Self-Perceived Skills in Communicating with Patient/Caregiver Regarding Unnecessary Tests		
Above average	11 (48%)	25 (47%)
Average	12 (53%)	27 (51%)
Below average	0 (0%)	1 (2%)

*Chi-square tests or Fisher exact tests demonstrated *p* > 0.05 for all comparisons of participant characteristics by workshop attendance

Over 97% of participants reported having prior experience communicating with patients/caregivers about unnecessary tests. Most self-rated their comfort and skill level with such communication scenarios as average or above average (Table 2), and 70% reported having received prior feedback on their communications skills with patients/caregivers about unnecessary tests.

Workshop attendance resulted in better performance on the knowledge test (4.3 ± 1.9 vs. 3.1 ± 1.7 out of 8, *p* = 0.01), but not better performance on the communication skills assessment (4.1 ± 0.8 vs. 4.0 ± 0.9 out of 5, *p* = 0.56). With regards to specific communication components, residents performed better on providing clear recommendations (4.1 out of 5) as compared to eliciting patient/caregiver concerns (3.7 out of 5) (*p* < 0.01) (Table 3).

**Table 3:** Resident Performance on the Structured Assessment of Communication Skills

	Mean (SD)^a^	Intraclass Correlation Coefficients (ICC)
Overall assessment	4.0 (0.7)	0.496
Provided clear recommendations†	4.1 (0.8)	0.532
Elicited patient concerns†	3.7 (1.8)	0.574
Demonstrated empathy	4.1 (1.0)	0.384
Confirmed agreement with patient/caregiver	3.8 (0.9)	0.403
General communication skills	4.1 (0.8)	0.496

^a^Scores on a 5-point Likert scale where 1 = not done; 3 = Attempted, but incomplete or not always effective; 5 = Excellent, complete and done effectively

†Residents performed significantly better at providing clear recommendations as compared to eliciting patient/caregiver concerns (*p* < 0.01)

The Cronbach’s alpha for the overall communication skills assessment was 0.88, indicating good to excellent internal consistency. ICC values comparing dual rater assessments on aggregate means of the subdomains and overall scores demonstrated moderate agreement, ranging from 0.384 to 0.574.

In the mixed-effects analysis, both higher training level (*p* = 0.01) and higher knowledge assessment scores (*p* = 0.046) were independent positive correlates of better global ratings on communication skills assessment, after adjusting for gender, training program, workshop attendance, and self-reported prior feedback on communication skills (Table 4).

**Table 4:** Results of a mixed-effects linear regression analysis with five pre-specified variables of interest as potential predictors of overall performance on the structured assessment

Predictor	Adjusted coefficient (95% CI)	*p*-value
Gender	−0.134 (−0.438, 0.170)	0.38
Post-graduate training level	0.255 (0.053, 0.456)	0.01
Workshop attendance	0.005 (−0.340, 0.350)	0.97
Resource stewardship knowledge	0.084 (0.002, 0.166)	0.046
Prior feedback on resource stewardship communication skills	0.145 (−0.008, 0.298)	0.06

## Discussion

We taught IM and Pediatric residents how to communicate with patients/caregivers who are seeking unnecessary tests/treatments as a way to enhance education on resource stewardship. Despite incorporating known approaches to promote learning of effective communication skills like role-play and observing videotaped patient and physician encounters, [32] our workshop only improved resident knowledge but did not significantly improve communication skills.

The most logical explanation for our findings is that a single workshop may not be sufficient to improve communication skills specific to resource stewardship. Additional reinforcement of the communication framework in the clinical learning environment is likely required to ensure delayed skills retention. In addition, it is possible that residents who tend to be effective communicators would perform well irrespective of workshop attendance.

Our study helps to advance education on resource stewardship in several important ways. Beyond providing an example of an educational workshop to teach effective communication when patients/caregivers request unnecessary tests, our findings also identified specific educational gaps that require closer attention. Residents in our study performed less well at eliciting patient/caregiver concerns compared to other communication domains. Prior research has demonstrated the importance of eliciting patient concerns to promote effective physician-patient interactions and decrease patient-related complaints [3, 33–35]. Educators should therefore emphasize the importance of eliciting patient/caregiver concerns when discussing unnecessary tests and/or treatments.

Second, with the recent focus on competency-based medical education [13, 14], we developed a tool to support assessments of communication skills about unnecessary tests and/or treatments that could be adopted by other programs. The design of our scale was methodical and attended to issues of validity in its construction [36]. To represent our construct, we chose content items to measure using a communication framework developed by Choosing Wisely®, with additional input from experts, and subsequently checked response processes of our raters through rigorous training. With respect to internal structure, our scale demonstrated moderate inter-rater reliability and good internal consistency. Skills ratings also correlated with knowledge test performance, suggesting a relationship between these variables within the resource stewardship construct.

This study has limitations to note. Workshop attendance is strongly encouraged but not mandatory. Therefore, volunteer bias may have influenced the results, since residents who attended the workshop may have a higher level of intrinsic interest, knowledge or skills in resource stewardship than those who did not. Since we did not assess real interactions with patients, additional research is needed to determine whether skills demonstrated on the structured assessment translate into real-world practice. There may also be value in engaging patients to further refine the construct of the tool [37] and validating the knowledge and skills assessment tools in different settings.

## Conclusion

An interactive workshop can improve knowledge of resource stewardship, but improving communication skills with patients/caregivers about unnecessary testing may require additional training or reinforcement in the clinical learning environment. These teaching and assessment approaches can support the integration of resource stewardship education into medical education.

## Additional files

Additional File 1: Post-OSCE Resource Stewardship Knowledge Test with Correct Answers Bolded. Copy of post-OSCE resource stewardship knowledge test with correct answers bolded. (PDF 165 kb)

Additional File 2: Resource stewardship OSCE communication scenario for Internal Medicine residents. Copy of the resource stewardship OSCE communication scenario for Internal medicine residents. (PDF 217 kb)

Additional File 3: Resource stewardship OSCE communication scenario for Pediatric residents. Copy of the resource stewardship OSCE communication scenario for Pediatric residents. (PDF 260 kb)

Additional File 4: Rating scale used to assess resident communication skills. Copy of rating scale used to assess resident communication skills. (PDF 200 kb)

## Abbreviations

ICC: Intraclass correlation coefficients
IM: Internal medicine
MRI: Magnetic resonance imaging
OSCE: Objective structured clinical examination
SP: Standardized patient
UofT: University of Toronto
