# Supplementary material for: Communicating wisely: teaching residents to communicate effectively with patients and caregivers about unnecessary tests
Source: BMC Med Educ. 2017 Dec 11;17:248. doi: 10.1186/s12909-017-1086-x (PMC5725805; doi:10.1186/s12909-017-1086-x)
Supplement: Supplementary file 1 — Post-OSCE Resource Stewardship Knowledge Test with Correct Answers Bolded. Copy of post-OSCE resource stewardship knowledge test with correct answers bolded. (PDF 165 kb) [file 12909_2017_1086_MOESM1_ESM.pdf]

Additional File 1
Post-OSCE Resource Stewardship Knowledge Test with Correct Answers Bolded

1. Have you ever had to communicate with a patient or their family around unnecessary testing requests (i.e. a patient/family requesting a test that is not clinically indicated)?

   Yes (If yes, go to question 2)   No (If no, go to question 5)

2. How would you rate your own skills in communicating with patients and families about unnecessary testing requests during your clinical experience?

1	2	3	4	5
Unsatisfactory				Outstanding

3. How would you rate your comfort level in communicating with patients and families who are requesting unnecessary testing?

1	2	3	4	5
Very uncomfortable				Very comfortable

4. How often have you received feedback from a staff physician after they have directly observed you communicate with a patient/family who is requesting an unnecessary test?

1	2	3	4	5
Never	Rarely	Sometimes	Often	Always

5. In your clinical practice, list the three (3) most common types of healthcare resource overuse that you have encountered:
   1.____
   2.____
   3.____

6. In your clinical practice, list the three (3) most common harms associated with over testing that you have encountered:
   1.____
   2.____
   3.____

7. In your experience, what are three (3) ways that you have contributed to unnecessary use of healthcare resources:
   1.____
   2.____
   3.____

8. Define value in healthcare:
   <u>Quality/Cost</u>

9. Which of the following constitutes the three components of the Institute for Healthcare Improvement's Triple Aim framework?
   a. Patient safety, quality and value
   b. **Improved care at the individual level, improved population health, and best value for public health system resources**
   c. Improved access to care, patient safety, and quality
   d. Decreasing overuse of healthcare resources, decreasing overtreatment, decreasing testing

10. All of the following are true statements regarding the increase in healthcare spending, EXCEPT:
    a. The cost associated with inappropriate care makes up a proportion of the increase in healthcare spending
    b. Overtreatment makes up a large percentage of health care expenditures
    c. **The primary factor in increase healthcare spending is the aging population**
    d. Two of the areas of greatest increase in expenditures is imaging and tests

11. The primary goal of the Choosing Wisely® initiative is to ______________________:
    a. Ration healthcare resources
    b. Discourage patients from asking physicians for unnecessary tests
    c. **Help physicians and patients engage in conversation about overuse**
    d. Increase the uptake of underutilized evidence based tests

12. (Internal Medicine). Which of the following tests or procedures is NOT discussed in the Society of Hospital Medicine's Choosing Wisely® top five (5) list:
    a. Repetitive CBC and chemistry testing in the face of clinical and lab stability
    b**. Screening CT heads for elderly patients with non-specific symptoms**
    c. Continuous telemetry monitoring outside of the ICU without a protocol for discontinuation
    d. Red blood cell transfusions for arbitrary hemoglobin cutoffs

12. (Pediatrics) Which of the following tests or procedures is discussed in the American Academy of Pediatrics' Choosing Wisely® top five (5) list:
    a. Repetitive CBC and chemistry testing in the face of clinical and lab stability
    b. **CTs in the immediate evaluation of minor head injury**
    c. Continuous telemetry monitoring in patients with bronchiolitis
    d. Red blood cell transfusions for arbitrary hemoglobin cutoffs

13. The main principles that guide the creation of every society's Choosing Wisely Canada® list include all of the following EXCEPT:
    a. **Must be geared toward expensive tests or procedures**
    b. Must be geared toward tests or procedures within the specialty's portfolio
    c. Must have evidence to support them

    d. Must be geared toward frequent tests or procedures

14. Which of the following is a step in the conversation physicians should have with patients when the patient is asking for an unnecessary test:
    a. The physician should inform the patient of the cost to the healthcare system of ordering the requested test
    b. **The physician should provide written information to the patient regarding the benefits and risks of the requested test**
    c. The physician should inform the patient that they can help arrange an appointment with another physician who may be willing to order the requested test
    d. The physician should provide anecdotal evidence to the patient of a time when the requested test had negative consequences

15. (Internal Medicine) (Internal Medicine). A patient presents to your clinic with vertigo. After taking a detailed history and performing a detailed physical examination, you believe that her presentation is most consistent with benign positional vertigo, and that further imaging with an MRI is not indicated. The patient has requested that you order an MRI to further evaluate her symptoms. Which should your LEAST FAVOURABLE approach:
    a. Ask the patient what she is concerned about
    b. **Take a patient-centered approach and order the MRI because the risk of radiation exposure from an MRI is negligible**
    c. Provide a clear recommendation to the patient and explain to her that an MRI is not clinically indicated because her physical examination is normal
    d. Once you finish counseling the patients, confirm that she agrees with the plan

15. (Pediatrics) A patient presents to your clinic with their parents who are requesting a screening panel (IgE testing) for food allergies. After taking a detailed history and performing a detailed physical examination, you believe that the child does not have any clinical symptoms to suggest food allergies and that such testing is not indicated. The parents have requested that you order testing. Which is the LEAST FAVOURABLE approach:
    a. Ask the parents what they are concerned about
    b. Take a family-centered approach and order the testing because the side effects are negligible
    c. Provide a clear recommendation to the parents and explain that such testing is not clinically indicated because it is unlikely that the child has food allergies
    d. Once you finish counseling the parents, confirm that they agree with the plan
