# Supplementary material for: Communicating wisely: teaching residents to communicate effectively with patients and caregivers about unnecessary tests
Source: BMC Med Educ. 2017 Dec 11;17:248. doi: 10.1186/s12909-017-1086-x (PMC5725805; doi:10.1186/s12909-017-1086-x)
Supplement: Supplementary file 2 — Resource stewardship OSCE communication scenario for Internal Medicine residents. Copy of the resource stewardship OSCE communication scenario for Internal medicine residents. (PDF 217 kb) [file 12909_2017_1086_MOESM2_ESM.pdf]

Additional File 2
Resource stewardship OSCE communication scenario for Internal medicine residents

Resource Stewardship Scenario – Instructions to residents at the time of their OSCE

Mrs. Fox is a 51 year-old patient that you are seeing in your General Internal Medicine clinic for follow up of her mild aortic stenosis. You last saw her one year ago. At that time, she had recently been discharged from the hospital. During her hospitalization (for cellulitis she developed after a cut on her lower leg) a transthoracic echocardiogram (TTE) was done because someone heard a murmur. The TTE showed mild aortic stenosis (AVA = 1.6cm2, PG = 20mmgHg), normal left ventricular size and function, and no other abnormalities. She was referred to your clinic for follow-up of her aortic stenosis.

Over the past year, since you last saw her, she states that she is doing well. Similar to last year, her exercise tolerance remains good. She has not experienced any chest pain, syncope or shortness of breath. She can walk, climb stairs, and play tennis without any difficulty. She has no other medical comorbidities and does not take any medications.

Her physical exam is unchanged compared to last year. Her HR is 80 and regular, and her BP is 135/85. She has a grade 1/6 systolic ejection murmur at the base that radiates to the clavicle and carotids. The remainder of her physical exam is entirely normal.

Your nursing staff informs you that she would like to talk to you about getting a repeat TTE to reassess her mild aortic stenosis.

You review the ACC/AHA Guidelines for appropriate use of TTE criteria, and see that for mild native valve aortic stenosis, routine surveillance with a repeat TTE should only be done every 3-5 years, when there has been no change in clinical status or cardiac exam.

Mrs. Fox is now waiting to see you to discuss getting a TTE for reassessment of her mild aortic stenosis. You are aware that this test is not indicated.

Resource Stewardship Scenario – Training instructions for standardized patients

Your name is Carla/Jim Fox. You are 51 years old. You live at home with your spouse. You have one child, a daughter, who is married and lives in Burlington. You have only been to the internal medicine clinic once before, a year ago. At your last visit you were following-up on a new diagnosis of mild aortic stenosis (this is a heart valve narrowing that may gradually progress and cause chest pain, shortness of breath, and fainting as it gets more severe) that was discovered during a brief hospitalization after a murmur was heard on your physical exam. The hospitalization was for an unrelated issue (the

unrelated issue was a skin infection – cellulitis – because of a cut on your lower right leg).

Background Medical History:
You are generally in very good health and do not take any prescribed or over the counter medications. You just take a multi-vitamin every day. You have not been hospitalized over the last year for any reason.

Further background if asked about (but not necessary for the resident to ask):
You are NOT having any symptoms and feel completely well. Specifically, if asked, you have never experienced any chest pain, or shortness of breath and you have never fainted. You remain very active. You can walk as long as you like, and you do not have any problems with stairs. You continue to play tennis twice a week when the weather is nice, without any problems.

You do not have any allergies, do not smoke, and drink alcohol very occasionally.

The purpose of your clinic appointment:
You are in clinic for a yearly follow-up because of your mild aortic stenosis (essentially the appointment with the physician is to check-in with you to ensure that you have not developed any symptoms). You have been a little bit anxious about this diagnosis, ever since it was found on the echocardiogram (ECHO) one year ago. You have been told that it is ok to continue with all of your usual activities, and you are aware of what symptoms to watch for (chest pain, shortness of breath, and fainting), of which you haven't experienced any, but just knowing about this heart valve issue makes you a bit anxious.

At this appointment, you are going to ask your physician to repeat the ECHO. You really want a repeat ECHO for peace of mind, since it has been a year since your previous ECHO. To make matters worse, your neighbour just needed a heart valve replacement surgery, after only being told a year ago about his 'leaky' valve (FYI note: a 'leaky' valve is likely to be a different diagnosis than yours – which is a 'narrow' valve). Hearing that story added to your anxiety about your own valve, and made you want a repeat ECHO even more.

Since you do not have symptoms, you are clinically stable, and your physical exam is unchanged from a year ago, there is no clinical reason to repeat your ECHO at this visit.

The goal of the scenario is for the resident to appropriately communicate with you why this ECHO is not necessary, and will not be done at this visit. You are not aware that the ECHO is not clinically indicated, and feel that is a reasonable request to have an ECHO done every year, given your heart condition.

The residents' performance should be assessed based on the scoring scheme for the Choosing Wisely® conversations rating scale. Your interaction should be guided by how he/she is doing in the scenario.

If the resident explains why the test is not necessary, describes the benefits and potential risks of the test, asks about your concerns, is empathic, and has good general communication skills, then you can remain calm. If they do all of the above, then calmly ask one more time "so you are sure I shouldn't have a repeat ECHO?" and then after any further explanation, accept that the test will not be performed.

If the resident is not clear in his or her explanation, does not talk about the risks and benefits, and does not convey why having the test is not appropriate, then you can get more anxious and upset in the manner in which you ask for the test.

PROMPTS:
Used to standardize the scenario and give all candidates an opportunity to discuss relevant issues.

Is there any downside to having a repeat ECHO?
• If the resident does not volunteer any downside, this prompt can be used.

I know other people, my neighbour, who needed valve surgery within one year of finding out about his condition.
• If the resident does not elicit your concerns about why you are so eager to have the test, you can volunteer this line.

Are you not giving me the test just to save the system money?
• This could be a prompt for all residents, even those that have explained the risks and benefits well.
