# Supplementary material for: Communicating wisely: teaching residents to communicate effectively with patients and caregivers about unnecessary tests
Source: BMC Med Educ. 2017 Dec 11;17:248. doi: 10.1186/s12909-017-1086-x (PMC5725805; doi:10.1186/s12909-017-1086-x)
Supplement: Supplementary file 3 — Resource stewardship OSCE communication scenario for Pediatric residents. Copy of the resource stewardship OSCE communication scenario for Pediatric residents. (PDF 260 kb) [file 12909_2017_1086_MOESM3_ESM.pdf]

Additional File 3
Resource stewardship OSCE communication scenario for Pediatric residents

Resource Stewardship Scenario – Instructions to residents at the time of their OSCE

Jonathan is a 2 year old boy who presents to the emergency department accompanied by his parents with a first episode of seizure.

Jonathan is a previously well, developmentally appropriate child with no significant past medical history or family history. Over the past 2 days, he has displayed typical signs of a viral upper respiratory tract infection. Today, he experienced a generalized tonic clonic seizure lasting 2 minutes. His temperature at this time was 39 C. He was then brought to the emergency department.

On examination, Jonathan was well appearing and quietly sitting next to his parents. His vital signs are within normal limits and he is afebrile (following ibuprofen). A full physical examination, including neurological examination, is normal. Jonathan is now playing in the waiting room with one of his parents.

You and the emergency department staff physician have diagnosed Jonathan with a simple febrile seizure.

Counsel Jonathan's parent Jamie regarding the diagnosis and management plan.

Resource Stewardship Scenario – Training instructions for standardized patients

Your name is Jamie Spence. You are 35 years old. You live at home with your spouse and 2 year old son Jonathan. Jonathan has had a cold for the past few days and today a seizure that lasted for 2 minutes and then stopped on its own. Jonathan's 'cold' consists of a cough, runny nose, and a fever. The seizure was shaking of his whole body and was very scary. He was a little out of it immediately after the seizure but has since returned to his usual behaviour.

In the past, Jonathan has generally been healthy. He has a few colds a year but no major illnesses. He has never had a seizure before. He isn't on any medications now other than Tylenol once or twice a day. He is growing and developing normally.

<u>The purpose of your emergency room visit:</u>

The initial purpose was to seek medical care following the seizure. Jonathan has been seen and examined and you are happy that the doctors have said that he doesn't need any medication or treatment. However, you do not agree with the doctors when they say that he doesn't need any tests to make sure the seizure was just a 'simple febrile seizure'. You would prefer that a MRI of the head be done just to make sure there is nothing wrong with Jonathan's brain. You keep thinking about a child in your neighborhood who had seizures and was diagnosed with bleeding in the brain. You want to be certain that Jonathan does not have this too.

Since Jonathan was diagnosed with a simple febrile seizure, imaging of the head (CT or MRI) is not indicated.

The goal of the scenario is for the resident to appropriately communicate with you why imaging of the head is not necessary and no further diagnostic tests are indicated. You are aware that head imaging is not indicated but feel that is a reasonable request as it will allow you to be absolutely sure that Jonathan's brain is ok.

The residents' performance should be assessed based on the scoring scheme for the Choosing Wisely® conversations rating scale. Your interaction should be guided by how he/she is doing in the scenario.

If the resident explains why head imaging is not necessary, describes the lack of benefits (imaging won't provide any information because the diagnosis is a clear) and potential risks (radiation with a CT, risk of anesthesia with MRI) of head imaging, asks about your concerns, is empathic, and has good general communication skills, then you can remain calm. If they do all of the above, then calmly ask one more time "so you are sure we can't get a MRI?" and then after any further explanation, accept that head imaging will not be ordered.

If the resident is not clear in his or her explanation, does not talk about the risks and benefits, and does not convey why head imaging is not appropriate, then you can get more anxious and upset in the manner in which you ask for an MRI.

<u>PROMPTS:</u>
Used to standardize the scenario and give all candidates an opportunity to discuss relevant issues.

<u>Is there any downside to an MRI?</u>
• If the resident does not volunteer any downside, this prompt can be used.

Well he's had colds and fevers before, how come he hasn't had a seizure with those episodes – surely something else must be going on now?

• If the resident does not elicit your concerns about why you are so eager to have the imaging, you can volunteer these lines.

Are you not giving him the MRI just because you don't want to? Because it's expensive?
This could be a prompt for all residents, even those that have explained the risks and benefits well.
