# Supplementary material for: Communicating wisely: teaching residents to communicate effectively with patients and caregivers about unnecessary tests
Source: BMC Med Educ. 2017 Dec 11;17:248. doi: 10.1186/s12909-017-1086-x (PMC5725805; doi:10.1186/s12909-017-1086-x)
Supplement: Supplementary file 4 — Rating scale used to assess resident communication skills. Copy of rating scale used to assess resident communication skills. (PDF 200 kb) [file 12909_2017_1086_MOESM4_ESM.pdf]

Additional File 4
Rating scale used to assess resident communication skills

CRITERIA FOR COUNSELLING PATIENTS/CAREGIVERS REGARDING UNNECESSARY TESTS

	1 (Not done)		2 (Attempted, but incomplete or not always effective)		3 (Excellent complete and done effectively)
**1. CLEAR RECOMMENDATIONS**					
• Explained why ordering the test is not necessary	1		2		3
• Described benefits and potential risks of the test	1		2		3
• Used clear language and avoided medical jargon	1		2		3
• Referred to evidence that supports the recommendation	1		2		3
**Overall Impression on Providing Clear Recommendations**	**1**	**2**	**3**	**4**	**5**
**2. ELICIT PATIENT/CAREGIVER CONCERNS**					
• Asked about my concerns (that make me want the test)	1		2		3
• Commented on non-verbal cues that indicate that I have concerns	1		2		3
**Overall Impression on Eliciting Patient/Caregiver Concerns**	**1**	**2**	**3**	**4**	**5**
**3. EMPATHY**					
• Told me that my concerns were understandable	1		2		3
• Allowed me time to express my concerns	1		2		3
• Told me that my emotional reaction was understandable	1		2		3
**Overall Impression on Empathy**	**1**	**2**	**3**	**4**	**5**
**4. CONFIRM AGREEMENT**					
• Summarized management plan briefly	1		2		3
• Offered me the opportunity to ask additional questions	1		2		3
• Confirmed with me my agreement with the treatment plan	1		2		3
• Offered a follow-up appointment to re-visit concerns	1		2		3
• Provided a clear description of symptoms/red flags that should alert an earlier follow-up	1		2		3
**Overall Impression on Confirming Agreement with Patient**	**1**	**2**	**3**	**4**	**5**
**5. GENERAL COMMUNICATION SKILLS**					
• Degree of coherence in the interview	1		2		3
• Verbal expression	1		2		3
• Non-verbal expression - N/A	1		2		3
• Responding to my needs	1		2		3
• Checked for my understanding of the information he/she provided	1		2		3

Figure 2. Rating scale used to assess resident communication skills (continued)

**Overall Impression on General Communication Skills**	**1**	**2**	**3**	**4**	**5**
**Overall Assessment of Choosing Wisely® Communication**	**1**	**2**	**3**	**4**	**5**

Did the resident order the test for you? (please circle one): YES NO
